# Predicting the suitable habitats of parasitic desert species based on a niche model with *Haloxylon ammodendron* and *Cistanche deserticola* as examples

**DOI:** 10.1002/ece3.8340

**Published:** 2021-12-12

**Authors:** Ping He, Yunfeng Li, Ning Xu, Cheng Peng, Fanyun Meng

**Affiliations:** ^1^ Beijing Key lab of Traditional Chinese Medicine Protection and Utilization Faculty of Geographical Science Beijing Normal University Beijing China; ^2^ Engineering Research Center of Natural Medicine Ministry of Education Faculty of Geographical Science Beijing Normal University Beijing China; ^3^ Key Laboratory of research and development of traditional Chinese medicine in Hebei Province Department of traditional Chinese Medicine Chengde Medical College Chengde China; ^4^ School of pharmacy Chengdu University of TCM Chengdu China

**Keywords:** *C. deserticola*, *H. ammodendron*, hotspot conservation, MaxEnt, parasitic species, suitable habitat

## Abstract

*Haloxylon ammodendron*, an excellent tree species for sand fixation and afforestation in the desert areas of western China, is threatened by climate change and anthropogenic activities. The suitable habitat of this species is shrinking at a remarkable rate, although conservation measures have been implemented. *Cistanche deserticola* is an entirely parasitic herb that occurs in deserts, is a source of “desert ginseng” worldwide, and has extremely high medicinal value. Little is known about using niche models to simulate habitat suitability and evaluate important environmental variables related to parasitic species. In this study, we modeled the current suitable habitat of *H*. *ammodendron* and *C*. *deserticola* by MaxEnt based on occurrence record data of the distributions of these two species in China. We grouped *H*. *ammodendron* and *C*. *deserticola* into three groups according to the characteristics of parasitic species and modeled them with environmental factors. The results showed that bioclimate was the most important environmental parameter affecting the *H*. *ammodendron* and *C*. *deserticola* distribution. Precipitations, such as annual precipitation, precipitation seasonality, and precipitation in the driest quarter, were identified as the most critical parameters. The slope, diurnal temperature range, water vapor pressure, ground‐frost frequency, and solar radiation also substantially contributed to the distribution of the two species. The proportions of the most suitable areas for Groups 1, 2, and 3 were 1.2%, 1.3%, and 1.7%, respectively, in China. When combined with cultural geography, five hot spot conservation areas were determined within the distribution of *H*. *ammodendron* and *C*. *deserticola*. The comprehensive analysis indicated that by using MaxEnt to model the suitable habitat of parasitic species, we further improved the accuracy of the prediction and coupled the error of the distribution of a single species. This study provides a useful reference for the protection of *H*. *ammodendron* forests and the management of *C*. *deserticola* plantations.

## Graphical abstract

Considering their parasitic characteristics of two species, we used niche model to predict the potential geographical distributions of *Haloxylon ammodendron* and *Cistanche deserticola* accurately. This study is the first to use a species distribution model to simulate the habitat suitability of parasitic species. We also designated hot spot conservation areas for *H. ammodendron* and *C. deserticola* and proposing conservation measures.

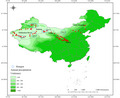

## 1 INTRODUCTION

*Haloxylon ammodendron*, an ultraxerophytic small tree of Chenopodiaceae, is widely distributed in Xinjiang, Inner Mongolia, Ningxia, Gansu, and other regions of China (Zhang, 2000; Zhang et al., 2010). *Haloxylon ammodendron* is a rare and endangered first‐class protected plant in Inner Mongolia left over from the Tertiary (Liu, 2017). As an excellent windproof and sand‐fixing plant, *H*. *ammodendron* plays a significant role in desertification control with strong drought, cold, and salt‐alkali tolerance (Xu & Li, 2008; Zhuang & Zhao, 2017). However, China's deserts are growing at a rate of 1300 square miles a year (Haner et al., 2016). *Haloxylon ammodendron* is distributed in extreme continental areas that have an annual average temperature of 2–11°C and annual precipitation of 30–200 mm or lower (Bai et al., 2008; Hu et al., 2011; Huang et al., 2003). Under drought stress, Verbascum glycosides, betaine, and soluble sugar show a linear change or a normal distribution with changes in temperature and precipitation (Zhang, 2018; Zheng et al., 2006). *Cistanche* *deserticola* is a perennial and fully parasitic herb in Lydanaceae, and the host plant is *H*. *ammodendron*, which means that its habitat is similar to that of *H*. *ammodendron* (Guo et al., 2005; Huang et al., 2012; Tan et al., 2004). As a secondary endangered medicinal plant, *C*. *deserticola* has a very high commercial market value with the functions of nourishing, immune enhancing, health care, aphrodisiac, and other functions (Liu, 2017; Song et al., 2008). Because *C*. *deserticola* cannot carry out photosynthesis, nutrients, and water must be obtained from the host, many studies (Huang, 1979; Huang et al., 1983; Wei, 1991; Yang, 1991) have shown that after being parasitized by *C*. *deserticola*, the relative water content of the assimilation branches of *H*. *ammodendron* decreases and that of *C*. *deserticola* increases significantly. For *H*. *ammodendron*, the decline in soil moisture will decrease the water potential and break the metabolic balance of the two species (Guo et al., 2004). By measuring the content of glucose, fructose, sucrose, etc., in different developmental stages of *H*. *ammodendron* and *C*. *deserticola*, Zheng et al. (2006b) concluded that the parasitism of *C*. *deserticola* greatly affects sugar metabolism, and this change is reflected in the amount of transfer. Zhao et al. (2019) analyzed eight different localities with *C*. *deserticola* in Inner Mongolia, Ningxia, and Xinjiang and found that the content of mullein glycosides and soluble polysaccharides was higher in Inner Mongolia, while the content of betaine was the highest in Xinjiang. Therefore, environmental conditions have an tremendous influence on *H*. *ammodendron* and *C*. *deserticola*, and it is of great significance to explore the effects of environmental factors on the two species.

Parasitic species include holoparasites and hemiparasites. Holoparasitic species rely entirely on hosts for nutrient transmission, while hemiparasitic species rely on their own metabolism for growth in addition to relying on hosts (Li, 2019; Nickrent, 2002). This characteristic divides parasitic species into benign and nonbenign parasites based on measuring the degree of absorption of the species by the host (Huang, Guan, Li, 2011; Huang, Guo, et al., 2011). Stewart and Press (1990) found that the parasitization of sorghum and sugarcane by *Striga* greatly reduced or even completely destroyed crop yields. Bao et al. (2015) discovered that after *Pedicularis equisetifolia* parasitizes a large number of Rosaceae, Leguminosae, and Gramineae plants, it improves the species diversity of pasture communities by adjusting the interspecific competition and balance among species in the same region. As a traditional Chinese medicinal material, *C*. *deserticola* is believed by practitioners to have the functions of nourishing kidney yang, the soul, and blood; decreasing Alzheimer's disease, fatigue, and aging; strengthening body immunity and memory; etc. (Guo, 2017; Hou et al., 2020). *Cistanche deserticola* has become a primary source of revenue for local residents after successful artificial parasitism in the 1980s (Liu & Yang, 2001; Tu et al., 2011). Many studies have shown that the betaine and Verbascum glycosides of *C*. *deserticola* have a strong linear relationship with *H*. *ammodendron*, which affects the metabolism and photosynthesis of *H*. *ammodendron*, so the leaves turn yellow or even deteriorate (Guo et al., 2005; Wang et al., 2017; Zhou, 2007). With this change, the water supply in *H*. *ammodendron* during the dormancy period is insufficient, which leads to fruit wrinkling or even reduces seed quality during the germination period (Hu et al., 2021; Lü et al., 2019). The N:P ratio of *H*. *ammodendron* leaves can be used as an indicator of growth nutrient supply status (Dong, 1996; Li et al., 2010). Huang, Guan, Li (2011), Huang, Guo, et al. (2011) compared the stoichiometric characteristics of C, N, and P in *H*. *ammodendron* leaves with those in soil in different habitats, and the results showed that the N:P ratio of *H*. *ammodendron* leaves was not affected by the content of C, N, and P in the soil, but the N and P contents were transferred between *H*. *ammodendron* and *C*. *deserticola* (Li et al., 2014). Therefore, environmental factors affect the normal development of *C*. *deserticola* by affecting the growth of *H*. *ammodendron*, and precipitation plays a key role in this process. Guo et al. (2005) studied the effects of inoculation of *C*. *deserticola* on *H*. *ammodendron* and found that after inoculation, the total loss of understory organisms was more than 10%, while the loss of seedlings was more than 40%; in addition, the number and years of *C*. *deserticola* inoculation greatly affected the *Haloxylon* forest (Wang et al., 2018; Zhang & Zhou, 2013; Zheng et al., 2019). Therefore, the metabolism and physiological characteristics of the *H*. *ammodendron* shadow environment affect the growth and quality of *C*. *deserticola*; in contrast, due to parasitic effects, *C*. *deserticola* has a great impact on *H*. *ammodendron*. Using niche models to simulate the habitat suitability of a single species may result in a large number of errors, and the effects of parasitic characteristics should not be overlooked. For *H*. *ammodendron* and *C*. *deserticola*, assessing the suitable habitat and distribution of them, understanding the impact of climate for two species, and exploring the influence of environmental factors on two species’ distribution are very necessary.

The unique habitat of *H*. *ammodendron* formed a unique relationship with nomadic cultures and their important livestock sources: camels. A large number of studies have shown that camels play a crucial role in the origin and development of many nomadic people, especially Mongolians (Bahxia, 2016; Li & Huang, 2020; Ma, 2019; Paul, 2016). The characteristics of enduring hunger and thirst and being resistant to heat and cold winters make camels better adapted to the natural geographical environment of the Gobi region (Bai, 2002; Hare, 2014). The Book of Han* XiongNu states "animal husbandry with water and grass," which is the way of life of ancient nomads and a significant feature of the ancient Silk Road (Fishman & Shirasu, 2021; Liang, 2020). Camels are not only a good means of transportation that play a prominent role in migration but also a key source of food that has a high amount of calories and is rich in protein (Lu et al., 2019; Yang et al., 2018; Yin, 2018). While gnawing on *H*. *ammodendron*, camels prune the branches and leaves, reducing the demand for nutrients (Bao, 2017). On the one hand, camels provide nutrition for *H*. *ammodendron* through defecation, but on the other hand, they expel undigested *H*. *ammodendron* seeds to promote reproduction (Wu, 2015). Camels contributed greatly to the ancient Silk Road based on extensive archeological studies, as they greatly reduced manpower, material, and financial resource consumption (Chang, 2018; Gao, 2019; Liang, 2020; Qi, 2004; Shi, 2016). Currently, “one route of the One Belt One Road” sets out from Beijing, passes through Xi'an, travels along the Gansu region to Urumqi, and finally reaches Afghanistan, which is similar to the path of the Silk Road (Liu, 2019a; Zeng, 2016). The economic mode and cultural exchange of nomads have gradually begun to change. The commercial market value of *C*. *deserticola* has rapidly increased because of its medicine value, resulting in deterioration of *C*. *deserticola* habitat when combing with strong climate changes (Tan et al., 2004). In addition, the excessive demand for *C*. *deserticola* neglects the protection of *H*. *ammodendron*, resulting in the degradation of the habitat for *H*. *ammodendron* (Tan et al., 2004; Wang et al., 2018). Therefore, it is very important to incorporate cultural and geographical factors into the study of species distribution to promote the virtuous circle of *H*. *ammodendron* and *C*. *deserticola*, as well as the development of the local agricultural economy. It is crucial to obtain the best suitable habitats and identify priority protection areas for the two species to promote conservation benefits.

A species distribution model (SDM) is a tool that has been gradually applied to the study of species distributions and the evaluation of significant parameters (Adhikari et al., 2019; Feeley & Silman, 2011). The CLIMEX, DOMAIN, GARP, and MaxEnt models have been widely used to assess endangered and invasive species (Dibyendu et al., 2019; Park et al., 2014; Peterson et al., 2007; Poutsma et al., 2008; Yang et al., 2020; Zhang et al., 2020). The MaxEnt model has been widely used to predict species habitats in recent years due to its easy operation, good performance, and high simulation precision (Borthakur et al., 2018; Schmidt et al., 2020; Singh et al., 2020; Wei et al., 2018; Zeng et al., 2016). Huang et al. (2020) used the MaxEnt model to predict the potential geographic distribution of American ginseng under different climate change scenarios in the Sanhe region. Zhang et al. (2018) simulated the potential geographic distribution of two peony species by using the MaxEnt model under climate change and assessed the potential expansion of peonies in the future. The key environmental variables of the two peonies were temperature seasonality, isothermality, and WET. Species distribution models are widely used in the simulation of various species, but it is very rare to simulate the distribution of parasitic species. Therefore, it is of great significance to use species distribution models to explore the habitat suitability of parasitic species.

In this study, the habitat suitability of *H*. *ammodendron* and *C*. *deserticola*, including dominant environmental factors in desert areas, was modeled. To predict the potential geographical distributions of *H*. *ammodendron* and *C*. *deserticola* accurately, their parasitic characteristics were fully considered. This study is the first to use a species distribution model to simulate the habitat suitability of parasitic species. The objectives of this study include (1) exploring the potential suitability of the current habitat of *H*. *ammodendron* and *C*. *deserticola*; (2) identifying the limiting variables of *H*. *ammodendron* and *C*. *deserticola*; (3) exploring the contribution of *H*. *ammodendron* to nomads, including the formation and transmission of culture and economics; and (4) designating hotspot conservation areas for *H*. *ammodendron* and *C*. *deserticola* and proposing conservation measures.

## 2 MATERIALS AND METHODS

### 2.1 Occurrence collection

**FIGURE 1:**
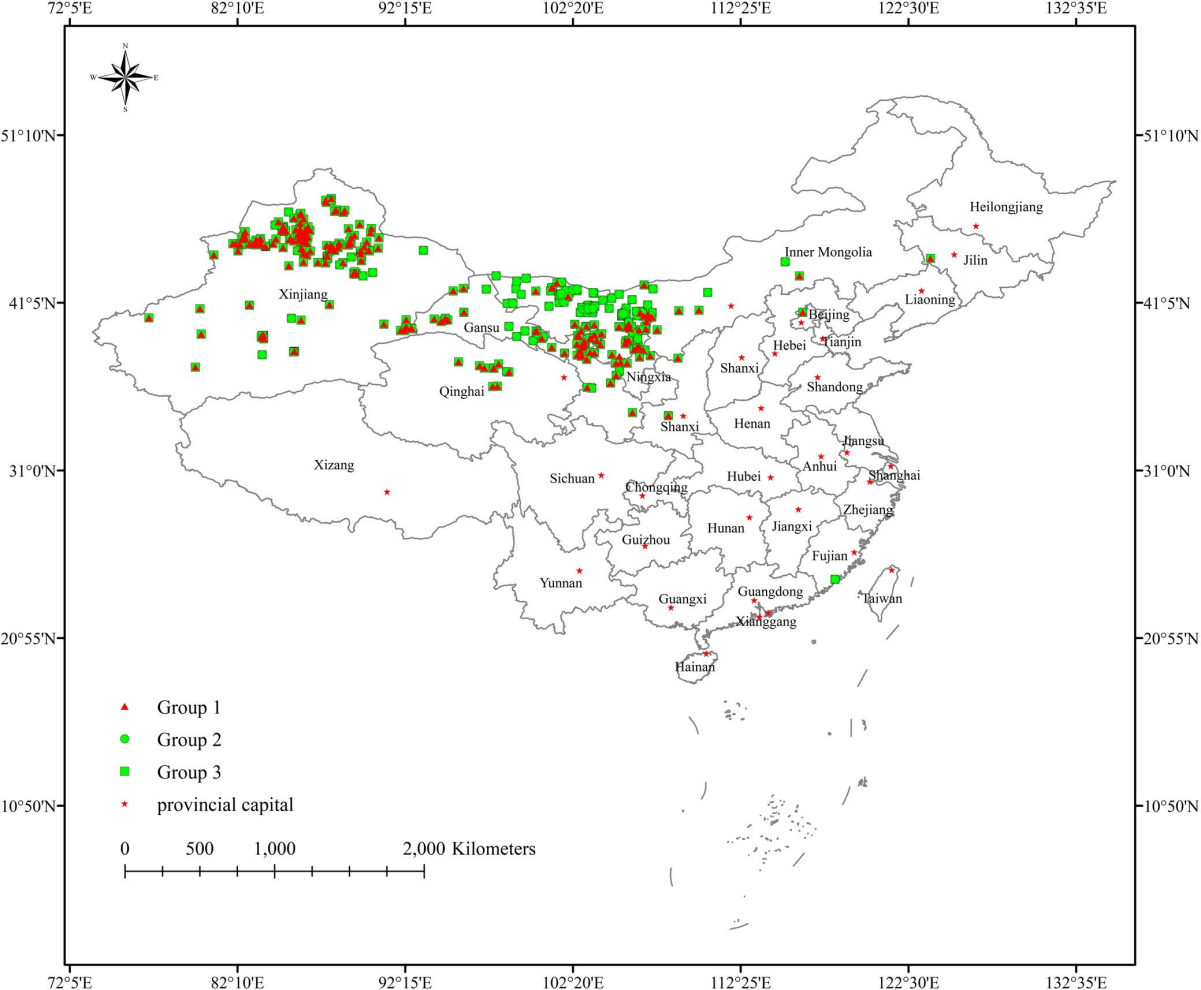
Distribution records of three groups, Group 1 is represented by a red triangle, Group 2 is represented by a green circle, and the green square is Group 3

To obtain accurate species distribution point data for *H*. *ammodendron* and *C*. *deserticola*, we conducted many searches of online databases, including the Global Biodiversity Information Facility (GBIF, https://www.gbif.org/), the Plant Photo Bank of China, (PPBC, http://ppbc.iplant.cn/), the Chinese Virtual Herbarium database (CVH, http://www.cvh.ac.cn/), the Specimen Resources Sharing Platform for Education (http://mnh.scu.edu.cn/), and the China National Knowledge Infrastructure (https://www.cnki.net/). For initial occurrence records, data with specific geocoordinates can be used directly while the data with study site descriptions need further processing. We removed the data on occurrence records with unspecific described geographical locations and utilized Google Earth (http://ditu.google.cn/) to determine the geocoordinates of occurrence records according to the specific described geographical locations. In accordance with the characteristics of *H*. *ammodendron* and *C*. *deserticola*, we divided the occurrence record data into three groups. We regarded the *H*. *ammodendron* occurrence data as Group 1 and the *C*. *deserticola* occurrence data as Group 2, and Group 3 was a coupling of Group 1 and Group 2. We regarded it as a new species’ occurrence data by putting the distribution point of *H*. *ammodendron* and *C*. *deserticola* into a set, and it was seen as Group 3. For three groups, duplicate records were deleted and filtered spatially to retain only one point in each grid cell (1 km×1 km) by using ArcGIS10.3. Finally, Group 1 had 271 distribution points, Group 2 had 154 distribution points, and Group 3 had 401 distribution points (Figure 1). All groups were saved in the “CSV” format as required by the MaxEnt model.

### 2.2 Environmental and sociocultural variables

We collected 32 environmental parameters that affected the distribution of *H*. *ammodendron* and *C*. *deserticola*. Nineteen bioclimatic variables, 2 terrain variables, and 1 solar radiation variable were obtained from the World Climate Database (www.worldclim.org). Among them, the 2 terrain variables were extracted from elevation variables containing slope and aspect. In addition, 4 soil texture factors were obtained from the National Cryosphere Desert Data Center (www.ncdc.ac.cn), including 0–30 cm clay (Clay 1), 30–60 cm clay (Clay 2), 0–30 cm sand (Sand 1), and 30–60 cm sand (Sand 2). Population density and land cover variables were obtained from the Socioeconomic Data and Applications Center (https://sedac.ciesin.columbia.edu/). Ground‐frost frequency (FRS), wet‐day frequency (WET), and vapor pressure (VP) were obtained from the IPCC database (http://www.ipcc‐data.org/obs/cru_ts2_1.html). To avoid overfitting in the final simulation results due to the high correlation between environmental factors, we used Pearson's correlations to filter 19 bioclimatic variables and 4 soil texture factors and eliminated one of the environmental variables with |*r*| ≥ 0.8 (Jayasinghe & Kumar, 2019; Phillips et al., 2006). We retained one environmental factor that was of high significance to the growth of *H*. *ammodendron* and *C*. *deserticola*. Therefore, the three groups with independent environmental variables and formats were all converted into “ASC” by using the ArcMap tool (Elith et al., 2010; Phillips, 2008).

Sociocultural variables were obtained from the geographical information system for the Silk Road (http://silkroad.fudan.edu.cn/project.html), the China National Knowledge Infrastructure (https://www.cnki.net/), and historical records. We deleted the inaccurate point data and compared the historical city with today's city to obtain accurate point information. Finally, we obtained information on 84 Silk Road distribution points (Figure 3).

### 2.3 Model operation and evaluation

We used MaxEnt 3.4.0 software (http://www.cs.princeton.edu/wschapire/Maxent/) to project the potential geographic distributions of Group 1, Group 2, and Group 3 with different distribution point information and environmental variables. Among them, precipitation and temperature are typically the most important variables that are considered to affect the distribution of species (Gao et al., 2018; Lobell & Gourdji, 2012). They affect the metabolic processes of vegetation. We also considered the impact of human activities on *H*. *ammodendron* and *C*. *deserticola*, so population density and land cover variables were used to evaluate their influence. For each group, 75% of the occurrence records were randomly used to train the model, and the remaining 25% were used to test the model. We used jackknife to test the importance of environmental variables and created response curves to measure how environmental variables affect *H*. *ammodendron* and *C*. *deserticola*. Finally, we used the average contribution rate of the three groups of environmental variables to evaluate the important environmental variables that affect *H*. *ammodendron* and *C*. *deserticola*. We ran the MaxEnt program with ten replicates to evaluate the averaged results, a logistic output format, a convergence threshold of 10^−5^, the maximum number of background points set to 10,000, and the maximum number of iterations set to 500. We used 1 as the regularization parameter value, and the output file type was “ASC.” The other parameters were software defaults, and outputs were generated using ArcGIS software.

To evaluate the accuracy of the model prediction, the area under the curve (AUC) and the kappa coefficient were used (Katz & Zellmer, 2018; Zhao et al., 2015). The area under the receiver operating characteristic (ROC) curve (AUC) was an effective method to evaluate model accuracy (Mas et al., 2013; Phillips, 2005). As another critical method, kappa statistics have been widely used in the study of species distribution and achieved good evaluation results (Chen, Du et al., 2015; Chen, Wu, et al., 2015; Duan et al., 2014; Landis & Koch, 1977). We used DIVA‐GIS 7.5 software (www.diva‐gis.org) to convert the prediction results into “GRD” format and build the corresponding stack dataset. Then, we used the same tool to split the occurrence data into multiple subsets and used the test set to test the prediction accuracy of our model. We created corresponding evaluation files and calculate ROC and kappa statistics with these. Generally, the higher the AUC value and kappa coefficient value were, the more accurate the model prediction (Townsend Peterson et al., 2007; Yang et al., 2019). We used these two parameters to comprehensively evaluate the accuracy of the three groups.

### 2.4 Economic and cultural characteristics

By consulting historical documents, we found that *H*. *ammodendron* is one of the main foods of *Camelus*. Moreover, nomads such as Mongolians use camels as a tool for economic and cultural exchanges with the Central Plains. At the same time, the formation of the Silk Road was closely related to camels, and many studies have shown that *H*. *ammodendron* was distributed along the ancient Silk Roads. We summarized the changes in the economic and cultural methods of nomads from ancient times to the present and found that there is a great correlation with *H*. *ammodendron*.

### 2.5 Priority protection area

To find the priority protection area for *H*. *ammodendron* and *C*. *deserticola*, we used ArcGIS 10.3 software to analyze the results of the model prediction and classify the suitability level into 4 levels by using manual classification. Three indicators were used to determine the conservation hot spot areas for *H*. *ammodendron* and *C*. *deserticola*. First, after modeling the current potential distribution of the two species, we overlaid Group 1 and Group 2 and compared them with Group 3 to obtain the priority areas for the cultivation of *H*. *ammodendron* and *C*. *deserticola*. Second, according to the ancient Silk Road route combined with the One Belt One Road, the results of coupling Group 1 and Group 2 were used to identify the hot spots. Finally, the habitat characteristics of *H*. *ammodendron* and *C*. *deserticola* were also considered, based on the most important environmental variable, which was assessed by the MaxEnt model.

## 3 RESULTS

### 3.1 Species distribution model and its accuracy

According to the average AUC and kappa for Group 1 to Group 3, the MaxEnt model performed excellently in predicting the distribution of these species. In general, the values of the AUC ranged from 0 to 1. We evaluated the accuracy of our model referring to the methods of some scholars (Du et al., 2018; Hu et al., 2014). When AUC>0.9, the modeling results were considered to reflect excellent model performance. An AUC value between 0.8 and 0.9 showed very good model performance; 0.7–0.8 indicated an average performance; 0.6–0.7 indicated a poor performance; and 0.5–0.6 indicated a very poor performance. We divided *H*. *ammodendron* and *C*. *deserticola* into three groups with different distribution data. We use kappa statistics, which can assess the consistency between the distribution data and the model prediction and can effectively reduce the imbalance between species distribution data (Brennan & Prediger, 1981; Cohen, 1960; Liao, 2011). A kappa value between 0.85 and 1 indicated that the model had an excellent performance, 0.7–0.85 indicated a good performance, 0.55–0.7 indicated a moderate performance, 0.5–0.55 indicated an average performance, and less than 0.4 indicated a failure (Allouche et al., 2006; Monserud & Leemans, 1992). The values were all calculated by using DIVA‐GIS 7.5 software, which is widely used in AUC and kappa statistics (Wang, Gen, et al., 2020; Wang, Yu, et al., 2020; Zhang et al., 2017). The larger the kappa and AUC values were, the higher the modeling ability of MaxEnt. For Group 1, AUC = 0.974, and kappa = 0.844; for Group 2, AUC = 0.973, and kappa = 0.865; and for Group 3, AUC = 0.955, and kappa = 0.794 (Table 1). This result indicates that the model performed well and generated high reliability.

**TABLE 1:** The AUC and kappa coefficient of the three groups

Group	AUC	KAPPA
Group 1	0.974	0.844
Group 2	0.973	0.865
Group 3	0.955	0.794

### 3.2 Potential suitable habitats for *H. ammodendron* and *C. deserticola*

**FIGURE 2:**
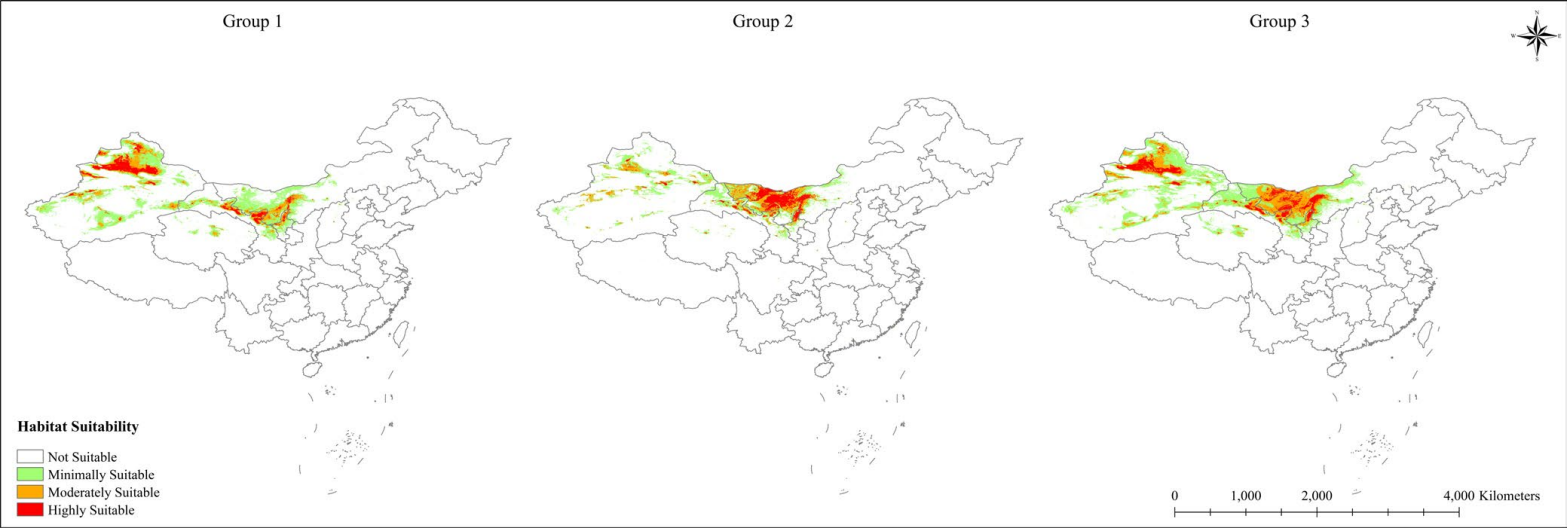
Current potential distribution of the three groups. Group 1 is *H*. *ammodendron*, Group 2 is *C*. *deserticola*, and Group 3 couples Group 1 and Group 2. The habitat suitability is highly suitable (red), moderately suitable (orange), minimally suitable (green), and not suitable (white) which was classified by ArcGIS 10.3

The suitable habitats were divided into four levels by using the reclassify tool of ArcMap 10.3 (Feng et al., 2021; Hu et al., 2014; Yang et al., 2013): highly suitable (0.6–1), moderately suitable (0.4–0.6), minimally suitable (0.2–0.4), and not suitable (0–0.2). For the three groups, the suitable areas of distribution were Group 3>Group 1>Group 2. We also determined that for each group, suitable areas were generally distributed in Xinjiang and Inner Mongolia, and minimally suitable areas were always 1–3 times higher than the highly suitable areas. Suitable areas for Group 1 were mainly distributed in Xinjiang, Group 2 was mainly in Inner Mongolia, and Group 3 was mainly in both provinces (Figure 2). However, for the highly suitable areas, Group 1 had the smallest suitable area, mainly distributed in Xinjiang, Gansu, and Inner Mongolia provinces with areas of approximately 1.24 × 10^5^ km^2^ and accounting for 1.2% of the area; Group 2 had a moderately sized suitable area, mainly distributed in Inner Mongolia, with sporadic distributions in Gansu and Xinjiang with areas of approximately 1.29 × 10^5^ km^2^ and accounting for 1.3% of the area; Group 3 included the most suitable area, mainly distributed in Xinjiang and Inner Mongolia, with sporadic distributions in Gansu and areas of 1.60 × 10^5^ km^2^, accounting for 1.7% of the area (Table 2).

**TABLE 2:** Area of potentially suitable habitat and its proportion for different groups

Suitable level	Group 1 (km^2^)	Portion (%)	Group 2 (km^2^)	Portion (%)	Group 3 (km^2^)	Portion (%)
Highly suitable	123,580.56	1.2	128,535.7	1.3	159,800	1.7
Moderately suitable	225,007.77	2.34	202,177.79	2.1	358,863.92	3.7
Minimally suitable	591,786.45	6.16	359,059.54	3.7	689,002.46	7.2
Not suitable	8,659,625.22	90.2	8,910,226.96	92.81	8,392,333.62	87.42

### 3.3 Important environmental variables

To obtain the important environmental variables that determined the distribution of *H*. *ammodendron* and *C*. *deserticola*, the jackknife test was used to evaluate the percentage of the contribution values of the three groups. We calculated the average contributions to comprehensively evaluate the importance of the environmental factors. The results reveal that the most dominant contributing environmental variable for *H*. *ammodendron* and *C*. *deserticola* is precipitation, with a 48% cumulative contribution, and this variable involved annual precipitation (BIO12, 32.86%), precipitation seasonality (BIO15, 8.4%), and precipitation of the driest quarter (BIO17, 6.73%). Then, slope (SLOPE, 7.43%), diurnal temperature range (TER, 4.9%), water vapor pressure (VP, 4.53%), ground‐frost frequency (FRS, 4.33%), and solar radiation (SRAD, 4%) also contributed greatly to the distribution of the species. Because *H*. *ammodendron* and *C*. *deserticola* are highly drought‐resistant and salt‐tolerant plants, they can survive under most environmental conditions. We chose the top eight environmental factors as the key variables affecting the growth and distribution of *H*. *ammodendron* and *C*. *deserticola*. For Group 1, variables including annual precipitation (BIO12, 32.3%), precipitation seasonality (BIO15, 15.8%), slope (SLOPE, 10.3%), diurnal temperature range (TER, 7.2%), annual temperature range (BIO7, 5.9%), ground‐frost frequency (FRS, 5.8%), water vapor pressure (VP, 4.5%), and annual mean temperature (BIO1, 4.1%) contributed substantially to the model. Annual precipitation (BIO12, 27.4%) was the most important variable affecting the distribution of Group 2, followed by the precipitation of the driest quarter (BIO17, 18.7%), wet‐day frequency (WET, 7.3%), water vapor pressure (VP, 6%), population density (DEN, 5.3%), diurnal temperature range (TER, 5.1%), slope (SLOPE, 4.6%), and temperature seasonality (BIO4, 4.2%). Group 3, which coupled Group 1 and Group 2, had great similarities with these two groups, which were influenced by annual precipitation (BIO12, 38.9%), slope (SLOPE, 7.4%), precipitation seasonality (BIO15, 7.2%), water vapor pressure (VP, 6.5%), annual mean temperature (BIO1, 6.3%), solar radiation (SRAD, 6%), annual temperature range (BIO7, 5.5%), and ground‐frost frequency (FRS, 5.5%). The key environmental variables and their percentage contributions for different groups are listed in Table 3.

**TABLE 3:** The key environmental variables and their percentage contributions for different groups

Group 1	Contribution (%)	Group 2	Contribution (%)	Group 3	Contribution (%)	Comprehensive	Contribution (%)
BIO12	32.3	BIO12	27.4	BIO12	38.9	BIO12	32.86
BIO15	15.8	BIO17	18.7	SLOPE	7.4	BIO15	8.4
SLOPE	10.3	WET	7.3	BIO15	7.2	SLOPE	7.43
TER	7.2	VP	6	VP	6.5	BIO17	6.73
BIO7	5.9	DEN	5.3	BIO1	6.3	TER	4.9
FRS	5.8	TER	5.1	SRAD	6	VP	4.53
VP	4.5	SLOPE	4.6	BIO7	5.5	FRS	4.33
BIO1	4.1	BIO4	4.2	FRS	5.5	SRAD	4

### 3.4 Mongolian culture and Silk Road culture

By consulting related documents such as ancient books, Silk Road archeology, and the Belt and Road Initiative, the results for Group 1 and Group 2 were overlaid by using ArcGIS tools. We found that the Book of Han* XiongNu states "animal husbandry with water and grass" to describe the lifestyle of nomads and the migration on the Silk Road at that time. In ancient China, hunting and grazing gave birth to the Mongolian culture, and the Mongolian people discovered that camels were a good tool and food source. *Haloxylon ammodendron* is one of the main foods of camels, and camel manure is a good fertilizer for *C*. *deserticola*. Bai (2002) found that when camels gnawed on the *H*. *ammodendron* tree, they did not damage the branches and eat them all, which achieved the effect of pruning branches and promoting nutrient circulation. Therefore, camels and *H*. *ammodendron* formed a chain‐type survival mode that effectively promoted the healthy development of ecology in desert and grassland areas. Because camels are large and extremely resistant to drought and hunger, they are widely used by people. On the one hand, people used camels as load‐carrying tools when migrating. At the same time, camels acted as an important medium for material and cultural exchanges between the caravans and the Central Plains, which led to the introduction of camels into the Central Plains, and the camel was gradually used as a transport tool when carrying. On the other hand, camels are an important source of food, and adult camels produce 250–350 kg of meat that is rich in protein and calories. Therefore, this characteristic has a great effect on its cold resistance, which gave birth to the Mongolian nation to a certain extent. However, camel resources were very scarce. Therefore, the number of camels owned by a family in ancient China often reflected the wealth of the family.

**FIGURE 3:**
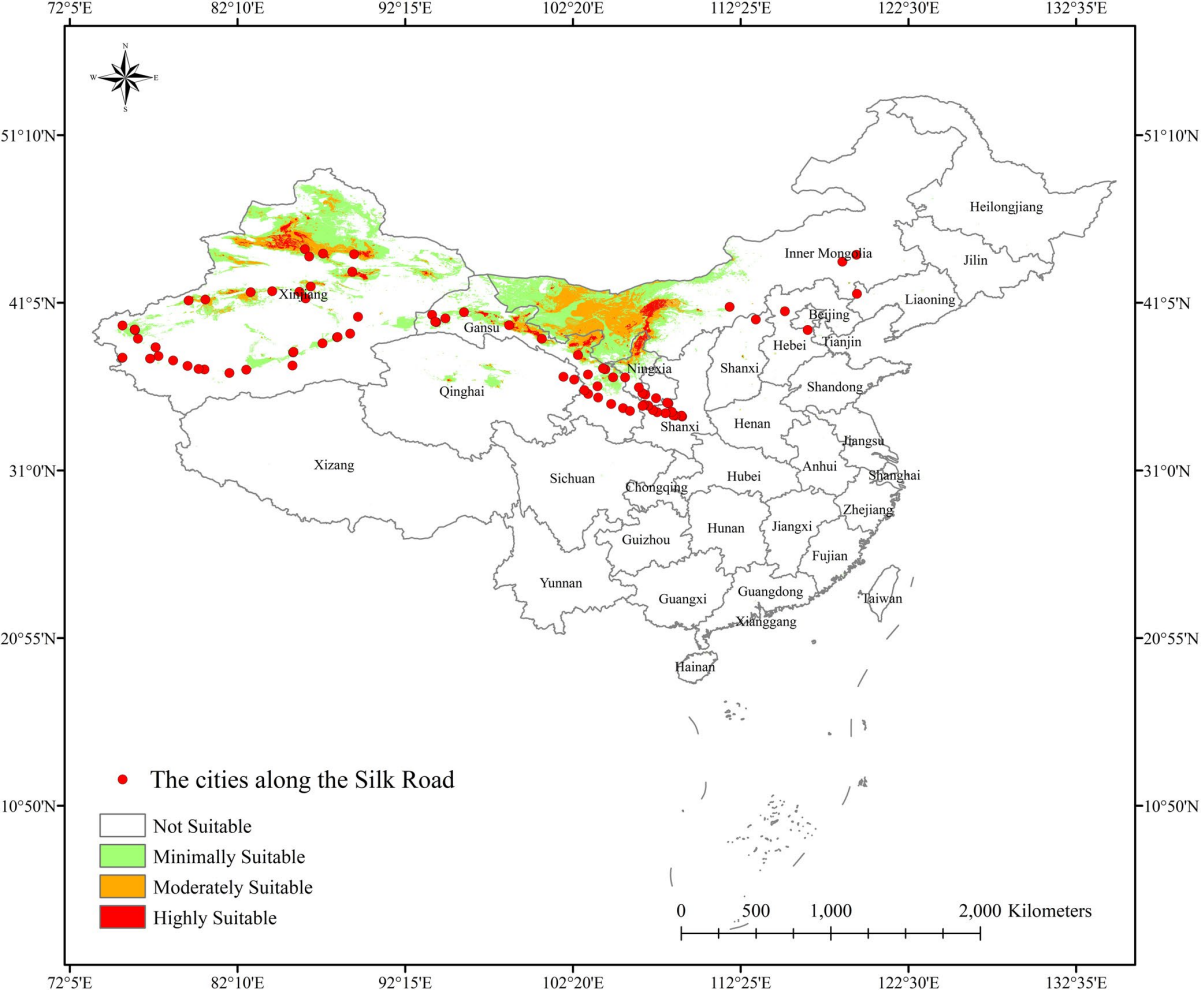
The Silk Road distribution points and the results of Group 1 and Group 2. In this picture, the results of habitat suitability were obtained by overlapping of Group 1 and Group 2

The opening of the Silk Road contributed greatly to communication between Chinese and Western civilizations. During the Han and Tang Dynasties, the Silk Road departed from Xian and reached Gansu in the west. It was divided into two lines, one passing through Urumqi and the other passing through the Hetian area. In the Yuan Dynasties, China's political center shifted to Beijing. On the one hand, the Silk Road departed from Beijing and Shangdu (in the Zhenglan Banner of the Xilin Gol League in the Inner Mongolia Autonomous Region) and passed through Inner Mongolia and Gansu to Xinjiang. On the other hand, the Silk Road departed from Beijing and passed through Shangdu to Russia. The freight transport used by caravans on the Silk Road was the camel, which played a substantial role in the Silk Road. Figure 3 shows that *H*. *ammodendron* and *C*. *deserticola* are mostly distributed along the Silk Road. Because of the existence of *H*. *ammodendron*, "camel culture" and "silk road culture" were born, thus strengthening the exchanges between Chinese and Western civilizations.

Economically, we found that during this period, the Mongolian people were mostly self‐sufficient due to grazing, and exchanging materials with Central Plains was based on camels. In terms of cultural communication, the transmission of culture was mostly carried out at the same time as economic exchanges.

### 3.5 Priority sites for *H. ammodendron* and *C. deserticola* conservation

**FIGURE 4:**
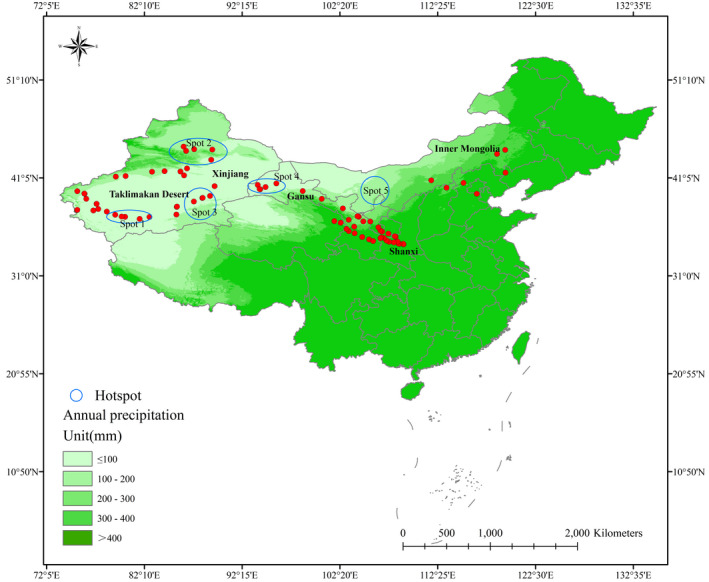
Hot spot map for *H*. *ammodendron* and *C*. *deserticola*. The order of the hot spot from left to right is Hot spot 1, Hot spot 2, Hot spot 3, Hot spot 4, and Hot spot 5

Through three indicators that were used to determine the hot spot conservation areas for *H*. *ammodendron* and *C*. *deserticola*, we developed a hot spot map. Figure 2 shows that the highly suitable area for *H*. *ammodendron* is more distributed in northwestern Xinjiang and less distributed in southwestern Inner Mongolia. The distribution of highly suitable habitat for *C*. *deserticola* was the opposite. The results of overlaying Group 1 and Group 2 show a great difference between the two sites, where most areas have changed from highly suitable areas to moderately suitable or minimally suitable areas. The coupling of Group 1 and Group 2 comprehensively fit the distribution of *H*. *ammodendron* and *C*. *deserticola*. This process excluded the errors caused by time differences and species parasitism. In Gansu and southern Xinjiang, the distribution of the two species was very stable, and Gansu was an area that not only the ancient Silk Road but also the Belt and Road passed through. As *H*. *ammodendron* has the effect of delaying desertification and *C*. *deserticola* is an important source of income for local residents, conservation hot spots were chosen comprehensively considering these factors. To facilitate the management and implementation of policies by local governments, we demarcated the conservation areas of *H*. *ammodendron* and *C*. *deserticola* with the city as the center (Figure 4).

Precipitation was the most important variable, so we also considered the habitat characteristics of the two species. Hot spot 1 is located in southwestern Xinjiang, which contains Hetian city. Wlumuqi, Tulufan, Kuitun, Jichang, Fukang, and Shihezi cities occur in Hot spot 2, which is located in western Xinjiang. For Hot spot 3, Kuerle city is the main area and is distributed on the southern edge of the Taklimakan Desert. All three hot spots are in Xinjiang province. Spot 4 is located in western Gansu Province, which contains Jiuquan and Dunhuang cities. For Hot spot 5, Wuhai, Shizuishan, Alashanzuoqi, and Yinchuan cities are critical areas for the distribution of *H*. *ammodendron* and *C*. *deserticola*.

## 4 DISCUSSION

As desert parasitic species, *H*. *ammodendron* and *C*. *deserticola* have extremely high economic value. *Haloxylon ammodendron* is a good drought‐resistant species with the characteristics of cold tolerance and drought tolerance (Brunel et al., 1995; Chesson et al., 2004). *Cistanche deserticola* is a traditional Chinese medicine that is highly valuable in nourishing the kidney, decreasing fatigue, and prolonging life (Chen et al., 2018; Li et al., 2016; Peng et al., 2016). This study used the MaxEnt model to simulate the potential geographic distribution of *H*. *ammodendron* and *C*. *deserticola* in China and divided them into three groups according to the parasitic features of the two species. The AUC value and kappa coefficient were used to evaluate the accuracy of the model (Allouche et al., 2006; Ben‐David, 2008; Brennan & Prediger, 1981). The values of the two evaluation methods indicated overall good performance of the model, and the model was adequate to determine the suitable habitat distribution of *H*. *ammodendron* and *C*. *deserticola* in China.

### 4.1 Key environment variables and ecological characteristics

The physiological nature of *H*. *ammodendron* and *C*. *deserticola* is determined by the habitat in which they live (Liu et al., 2019; Wei et al., 2007; Xu et al., 2014). Habitat formation for a particular species is often affected by bioclimate, altitude, topography, soil, etc., and human factors cannot be neglected because they are related to people with the greatest economic interest (Jeffrey et al., 2015; Liu et al., 1999). Our results show that precipitation was most important for the distribution of *H*. *ammodendron* and *C*. *deserticola*, which is different from the conclusion that temperature was a key variable (Xiao et al., 2019; Yang, 2017). Among the various variables adopted in the model, bioclimate factors were significant for the three groups.

Before the 1980s, *H*. *ammodendron* was widely distributed in northern China under natural conditions, and *C*. *deserticola* parasitized the roots of *H*. *ammodendron* (Tu et al., 2011). *H*. *ammodendron* is highly resistant to harsh environments and is extensively used for restoring degraded drylands (Li et al., 2013; Luo et al., 2017). The three groups and the comprehensive results show that precipitation has had an attenuating effect on the distribution of the two species. There is abundant evidence showing that water plays a significant role in the formation of polysaccharides in plants (Matveev et al., 2000; Sun, 2018; Zhang et al., 2003; Zhao, 1966). The water source of young *H*. *ammodendron* is mainly derived from precipitation until the roots reach sufficient depths, and deep soil water becomes a key supply model (Ma et al., 2007). When the water condition is very good, *H*. *ammodendron* can resist high temperatures with high transpiration; in contrast, this species can resist high temperatures and drought by low transpiration (Li, 1992). While the transpiration rate is under temperature stress, it is also greatly affected by water sources. The plant height, growth of branches and leaves, crown width, and biomass of *H*. *ammodendron* are mainly determined by water conditions (Imanuel, 1973; Li & Yang, 1996). Under desertification, osmotic adjustment plays a key role in determining the capacity of humidity (Song et al., 2006). Osmotic adjustment can result in the accumulation organic and inorganic solutes to decrease osmotic potential and will help plant metabolism (Blum, 2017; Hsiao et al., 1976). As important organic osmotic regulators, betaine and soluble sugars are essential to protecting *H*. *ammodendron*, helping it avoid drought stress (Lü et al., 2019; Yooyongwech et al., 2006). Zheng et al. (2006) compared the linear correlations in *H*. *ammodendron* and *C*. *deserticola* with soluble polysaccharides and betaine and concluded a counterbalance phenomenon. Furthermore, water affects the photosynthetic rate by affecting the stomatal conductance of leaves, and the amount of water affects the physiological metabolism of plants that produces sugar and alkalis (Cui, 2014; Tan, 2004). Therefore, moisture largely determines the growth and distribution of *H*. *ammodendron* and *C*. *deserticola*. Li et al. (2019) and Ma et al. (2017) also believe that the overall contribution rate of precipitation is higher than that of temperature, which is consistent with the results of our research.

In the wild, the seeds of *H*. *ammodendron* are scattered on the lower part of a windward slope or the bottom of a leeward slope under the wind direction (He et al., 2015; Wang et al., 2007). For Group 1 and Group 3, the slope was an important factor for *H*. *ammodendron*, but for Group 2, the opposite was true. Previous studies have shown that the afforestation land for *H*. *ammodendron* is usually fixed or semifixed sand dunes and the lower part of windward slopes or at the bottom of leeward slopes (Guo et al., 2020; Liu, 2019a). Ancient populations mostly adopted the planting method "seedlings go with the water," which is a good method for local residents (Liu, 2019b; Sun & Zhu, 2012). On the lower part of windward slopes or the bottom of leeward slopes, *H*. *ammodendron* is less affected by wind erosion, it easily forms a confluence during rainfall, and the groundwater level is high (Chen, Du et al., 2015; Chen, Wu, et al., 2015; Hu, 1984; Sun & Zhu, 2012; Zhou et al., 2019). Therefore, similar to wet‐day frequency, slope indirectly indicates the importance of precipitation to *H*. *ammodendron* and *C*. *deserticola*. In April and May, the florescence stage of *H*. *ammodendron* occurs, but its ovary is temporarily dormant during the dry summer until August (Huang et al., 2001). Between August and September, the rainy season and suitable temperatures will break the dormancy of the ovary, germination will begin, and seeds will ripen in this stage (Huang et al., 2001; Wang, 2006). Therefore, temperature and precipitation greatly affect the physiological and phenological periods of *H*. *ammodendron*, which will influence *C*. *deserticola* inoculation.

For Group 2, the distribution of *C*. *deserticola* was widely affected by human activities after the successful cultivation of *C*. *deserticola* (Tu et al., 2011). Therefore, population density and land cover variables were used in this study, but the former had good performance. In Group 3, this factor became nonsignificant, which indicates that the cultivation of *H*. *ammodendron* and *C*. *deserticola* needs further fitting under planning. For the three groups, the contribution rates of the environmental variables were slightly different, which was determined by the sensitivity of the two species to the environmental factors. To accurately assess the response of environmental variables to *H*. *ammodendron* and *C*. *deserticola*, it is necessary to select enough environmental parameters while considering local characteristics. A comprehensive assessment of environmental variables will facilitate the selection of *H*. *ammodendron* and *C*. *deserticola* hot spots, which will further couple the shortcomings of single‐species modeling.

### 4.2 Economic and cultural characteristics of nomads

In ancient China, *H*. *ammodendron* promoted the prosperity of the Silk Road by enabling camel reproduction (Chang, 2018; Liang, 2020). After the successful cultivation of *C*. *deserticola* in the 1980s, nomads’ economic sources and cultural communications began to change. Local residents have implemented the transformation of their economic mode mainly by planting *C*. *deserticola*, which is an outside‐in reformation and minimizes the damage to *H*. *ammodendron* forests. A new camel tourism culture was gradually born, converting cultural communications from input to output models, which is also an important financial resource. From *H*. *ammodendron* to *C*. *deserticola* or camels to ultimately people, an ecological cycle was formed. To strengthen future *H*. *ammodendron* and *C*. *deserticola* protection efforts, the differences between the three groups must be considered, and the economic and cultural characteristics of the region are also very significant reference indicators.

### 4.3 Priority conservation sites for *H. ammodendron* and *C. deserticola*

Known as “the oasis in the desert,” *H*. *ammodendron* is a major contributor to desert biomass, and there are four *Haloxylon* natural reserves in China: Ganjiahu *Haloxylon* Forest National Nature Reserve, Qitai Desert Grass Nature Reserve, Qaidam *Haloxylon* Forest National Nature Reserve, and Urad *Haloxylon* Forest Mongolian Wild Ass National Nature Reserve. The deterioration of the environment and human destruction threatens the sustainability of *H*. *ammodendron* and will further affect the distribution of *C*. *deserticola*. A large number of *H*. *ammodendron* and *C*. *deserticola* habitats have been destroyed, and desertification is increasing. By comparing the results for the three groups with the current distribution of the two species and combining the characteristics of cultural geography, these four nature reserves are also within the scope of the sites.

In Xinjiang and Inner Mongolia, the highly suitable distribution areas of the three groups are in great imbalance, and the collocation of the two species should be considered while cultivating *H*. *ammodendron* to achieve a balanced ecological effect. Paying more attention to the cultivation of *C*. *deserticola* in Hot spot 2 will have great significance, while in Hot spot 5, the protection of the *Haloxylon* forest will alleviate the grassland desertification. The distribution of *H*. *ammodendron* and *C*. *deserticola* in Gansu was not just a function of maintaining ecology but connecting Xinjiang with Inner Mongolia, and Group 3 accurately expressed this relationship. In the southern part of the Taklimakan Desert, suitable habitat for *H*. *ammodendron* is very sparse, so we suggest that the inoculation time for *C*. *deserticola* is no more than 10 years because parasitism will have adverse effects on *H*. *ammodendron* (Guo et al., 2011; Huang et al., 2009; Zheng et al., 2006b). All conservation of hot spots must be integrated with the local policies on *H*. *ammodendron* and *C*. *deserticola*. It is necessary to cover include *H*. *ammodendron* protection areas in a grid model with the city, county, and town as the center during urbanization.

## 5 CONCLUSIONS

**FIGURE A1:**
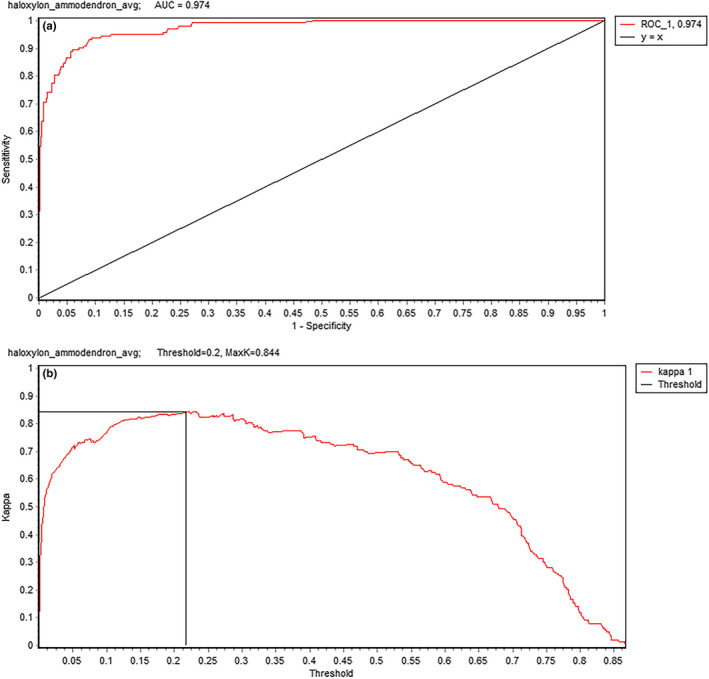
ROC curves (a) and Kappa Coefficient (b) of the Group 1 (*H*. *ammodendron*)

**FIGURE A2:**
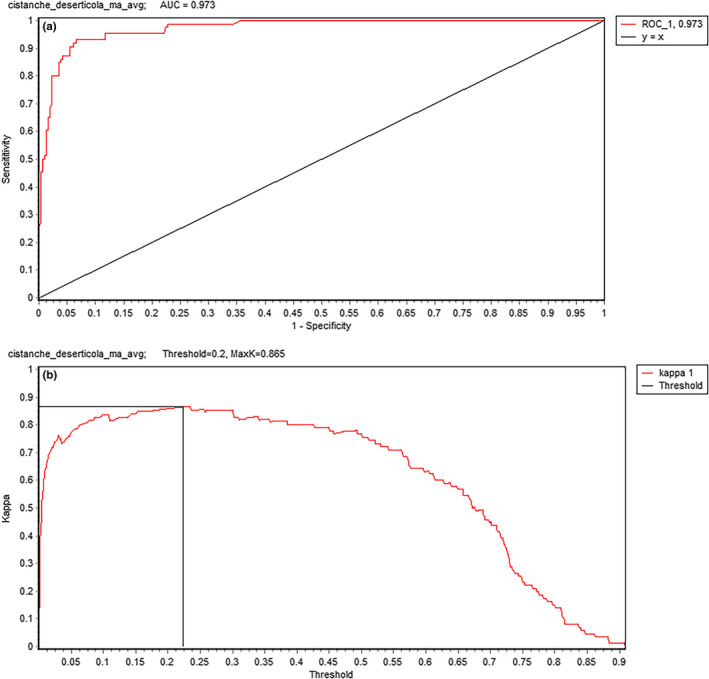
ROC curves (a) and Kappa Coefficient (b) of the Group 2 (*C*. *deserticola*)

**FIGURE A3:**
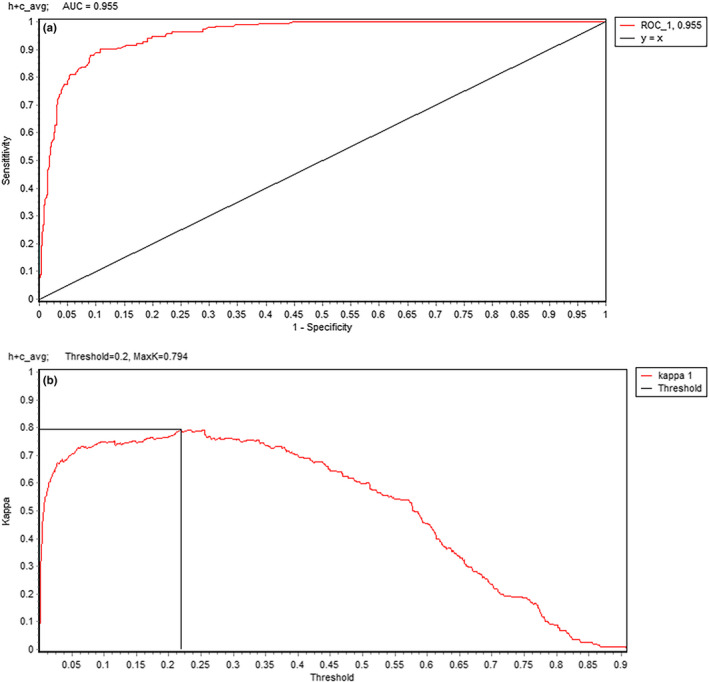
ROC curve (a) and Kappa Coefficient (b) of the Group 3 (*H*. *ammodendron* and *C*. *deserticola*)

Models of suitable habitat for *H*. *ammodendron* and *C*. *deserticola* with different environmental variables were successfully established in this study. These results indicate a great imbalance in the total suitable habitat of parasitic species. By coupling Group 1 and Group 2, the issues caused by *C*. *deserticola* parasitism were further diluted. Precipitation was considered the most critical factor with reliable results, which is different from previous research. We integrated human geography into this research, and it played a significant role in the choice of conservation areas. To further facilitate a reasonable management strategy, we divided five hot spot conservation areas based on comprehensive results, which will recoup the costs of protection efforts. These prediction results will be useful references for the management of *H*. *ammodendron* and the cultivation of *C*. *deserticola*, as well as a theoretical basis for desertification control. To optimize the modeling conclusions, more detailed work must be considered in future studies, such as the optimization of environmental parameters, the selection of sample size, and the coupling of multiple models. At the same time, the coupling of *H*. *ammodendron* and *C*. *deserticola* can eliminate the error caused by parasitic to a certain extent, but it is only a cursory estimate. Our future work will focus on more refined modeling, especially the parasitic characteristics and determination of sample size, to obtain more accurate results. (Figures A1, A2, A3).

## CONFLICT OF INTEREST

The authors declare no competing interests.

## AUTHOR CONTRIBUTIONS

**Ping He:** Conceptualization (lead); data curation (lead); formal analysis (lead); investigation (lead); methodology (lead); resources (lead); software (lead); supervision (lead); validation (lead); visualization (lead); writing–original draft (lead); writing–review and editing (lead). **Yunfeng Li:** Formal analysis (equal); funding acquisition (supporting). **Ning Xu:** Formal analysis (equal). **Fanyun Meng:** Data curation (equal); funding acquisition (equal); project administration (equal). **Cheng Peng:** Funding acquisition (equal); project administration (equal).

## Data Availability

Climate data and MaxEnt input files: https://datadryad.org/stash/share/TcrZRqFX‐G0CUJuVJ9vG_JkrLHA‐qITpOlyDrOq9l10.
